# Real-world outcomes of immunotherapy-based neoadjuvant therapy in resectable non-small cell lung cancer

**DOI:** 10.3389/fimmu.2023.1268251

**Published:** 2023-09-25

**Authors:** Jie Shen, Linping Gu, Yuwen Qi, Yaxian Yao, Shun Lu, Zhiwei Chen

**Affiliations:** Department of Shanghai Lung Cancer Center, Shanghai Chest Hospital, School of Medicine, Shanghai Jiao Tong University, Shanghai, China

**Keywords:** non-small cell lung cancer (NSCLC), neoadjuvant, immunotherapy, chemotherapy, clinical study

## Abstract

**Objectives:**

Recent clinical studies have demonstrated that immunotherapy-based neoadjuvant therapy have promising effectiveness for patients with resectable non-small cell lung cancer (NSCLC) in terms of pathologic response. Therefore, we performed this study to investigate whether immunotherapy-based neoadjuvant therapy is effective and safe for patients with resectable NSCLC.

**Materials and methods:**

This open-label observational two-arm clinical study was performed at Shanghai Chest Hospital in China with patients who had resectable NSCLC and received two to three cycles of immunotherapy-based neoadjuvant therapy or neoadjuvant chemotherapy alone, followed by surgical resection. The primary endpoint was a major pathologic response (MPR). The secondary endpoints include a complete pathological response (pCR), a radiologic response to neoadjuvant therapy (TRR), event-free survival (EFS), and overall survival (OS).

**Results:**

A total of 51 patients was included in this clinical study, of which 31 patients received immunotherapy-based neoadjuvant therapy and 20 patients received neoadjuvant chemotherapy alone. The percentage of patients achieving a major pathologic response was 41.9% with immunotherapy-based neoadjuvant therapy and 15.0% (95% CI, 0.008 to 0.468; P = 0.043) with neoadjuvant chemotherapy alone. The percentage of patients with pathologic complete response was 19.4% in the immunotherapy-based group and 5% (95% CI, -0.069 to 0.318; P = 0.223) in the chemotherapy group. The radiographic response rate was 71% after immunotherapy-based neoadjuvant therapy and 60% (95% CI, -0.143 to 0.359; P = 0.417) after neoadjuvant chemotherapy. At a median follow-up of 28 months, the median EFS and OS endpoints were not reached.

**Conclusions:**

Neoadjuvant immunotherapy offers a considerable advantage over chemotherapy alone for resectable NSCLC in terms of the major pathologic response. Moreover, it did not enhance the risk of adverse events or hinder surgical resection.

## 1 Introduction

Lung cancer ranked first among all cancer-related mortality worldwide in 2022 (1, 2). More than 80% of these cases are classified as non-small cell lung cancer (NSCLC), and approximately 20% to 25% of NSCLCs can be treated surgically (3). However, recurrence and death occur in 30% to 55% of patients who undergo surgical resection (4, 5). Studies have shown that the relative benefit of neoadjuvant chemotherapy compared with surgery alone is only 5% to 6% for 5-year recurrence-free survival and overall survival, which is not significant (6).

Neoadjuvant therapy aims to reduce tumor size and burden, leading to better surgical resection and better prognosis (7). Neoadjuvant immunotherapies act on both the tumor and the tumor microenvironment and are thus thought to be potentially more effective (8).

Recent phase II-III clinical studies have reported the feasibility and safety of neoadjuvant immunotherapy in resectable NSCLC. In a study of CheckMate-159, two cycles of nivolumab induced major pathological response (≤ 10% residual viable tumor, MPR) in 45% of patients, including 15% of complete pathological response (no residual viable tumor, pCR) (9). Two cycles of atezolizumab (LCMC3) resulted in 18% of patients achieving a major pathological response, with 5% achieving a complete pathological response (10). In a study of three cycles of nivolumab with chemotherapy (CheckMate-816), 36.9% of patients achieved a major pathological response, with 24% achieving a complete pathological response (11). Additionally, 4 cycles of atezolizumab with chemotherapy resulted in a major pathological response in 57% of patients, with 33% having a complete pathological response (12). In almost all clinical studies, the median EFS and OS endpoints were not met. MPR and pCR were used as surrogate endpoints in many clinical trials to evaluate efficacy. Several studies have demonstrated that the percentage of viable tumor cells in the resected tumor specimen correlates with OS and EFS in patients with NSCLC (13, 14).

To assess the efficacy and safety of neoadjuvant immunotherapy in a real-world NSCLC population, we conducted this observational study.

## 2 Materials and methods

### 2.1 Study design and patients

This open-label observational two-arm clinical study of neoadjuvant therapy in locally resectable NSCLC was performed at Shanghai Chest Hospital in China.

Patients were eligible for enrollment if they were ≥ 18 years of age and had an Eastern Cooperative Oncology Group performance status score of 0 or 1 (15). They should also have histologically or cytologically confirmed locally NSCLC of stages I to III (according to the staging criteria of the eighth edition of the American Joint Committee on Cancer) (16) that was determined to be surgically treatable by a multidisciplinary team. A whole-body bone scan, abdomen ultrasound, magnetic resonance imaging (MRI) of the brain, and contrast-enhanced computed tomography (CT) of the chest were used for staging. Nodal (N3) involvement had to be proven by lymph node sampling. Immunodeficiency, a history of autoimmune disease, or ongoing systemic immunosuppressive therapy were included in the exclusion criteria for patients. Patients with known EGFR mutations, ALK translocation or ROS-1 rearrangement were excluded. These three mutant genes, as well as PD-L1 expression levels (22C3 pharmDx kit), were assessed based on biopsy sample at diagnosis of NSCLC.

All included patients gave their informed consent to participate according to the Declaration of Helsinki. The study was approved by the Institutional Review Board of Shang Chest Hospital (No. KS1971).

### 2.2 Procedures

In this clinical study, two to three cycles of immunotherapy-based neoadjuvant therapy or neoadjuvant chemotherapy alone were administered to all eligible patients. Patients who received immunotherapy-based neoadjuvant therapy included patients who received immunotherapy alone and patients who received immunotherapy plus chemotherapy. We collectively referred to the patients who received neoadjuvant immunotherapy alone and those who received immunotherapy plus chemotherapy as the ‘immunotherapy group’, and we referred to the patients who received neoadjuvant chemotherapy alone as the ‘chemotherapy group’. The choice of chemotherapy regimen was made with reference to the guidelines of the National Comprehensive Cancer Network and the Chinese Society of Clinical Oncology. Patients in the immunotherapy group were treated with PD-1 inhibitor plus chemotherapy or immunotherapy alone. Patients received these drugs on day 1 of each 21-day cycle. PD-1 inhibitor included pembrolizumab (200mg), nivolumab (360mg), stintilimab (200mg), and tislelizumab (200mg). In neoadjuvant therapy, dose reduction was permitted for chemotherapy drugs but not for PD-1 inhibitors. Treatment could be interrupted or postponed if any intolerant toxicity occurred and could be resumed at any time if the conditions for restarting treatment were met. After two cycles of neoadjuvant therapy, contrast-enhanced CT was performed to assess the change in tumor size. Multidisciplinary teams would decide whether to proceed with another cycle of immunotherapy-based neoadjuvant therapy based on the change in tumor size, the adverse events during treatment, and the patients’ own wishes. Surgical resection was scheduled approximately four weeks after the final dosage of neoadjuvant therapy.

### 2.3 Endpoints and assessment

The primary endpoint was a major pathologic response (MPR), or ≤ 10% of residual viable tumor cells in the primary tumor. The key secondary endpoint was a complete pathologic response (pCR), or 0% of residual viable tumor cells in the primary tumor and sampled nodes. Other secondary endpoints included radiologic response to neoadjuvant therapy (TRR), event-free survival (EFS), and overall survival (OS). EFS and OS were calculated from the first dosage of neoadjuvant therapy.

Contrast-enhanced CT of the chest was done at least 7 d before neoadjuvant therapy began, and this was repeated within 7 d of surgery. Changes in tumor size were evaluated according to Response Evaluation Criteria in Solid Tumors (RECIST), version 1.1 (17).

To track whole blood cell counts and biochemical markers during neoadjuvant therapy, all patients received laboratory blood testing once a week. Adverse events and abnormal laboratory results were graded according to Common Terminology Criteria for Adverse Events, version 4.0.

Patients with surgical resection are being followed up every 3 months for the first 2-3 years, every 4-6 months for an additional 2 years, and annually thereafter. Follow-up assessments included whole blood cell, biochemical markers, tumor marker, whole-body bone scan, abdomen ultrasound, magnetic resonance imaging (MRI) of the brain, and contrast-enhanced computed tomography (CT) of the chest. The database of EFS and OS analyses was locked on October 31, 2022 (median follow-up, 28 months).

### 2.4 Statistics

Data were expressed as median and range unless otherwise indicated. *P*  < 0.05 (two-sided) was regarded as statistically significant. SPSS software (IBM SPSS Statistics 26) and R version 4.2.2 were used to perform all statistical analyses.

## 3 Results

### 3.1 Patients

**Figure 1:**
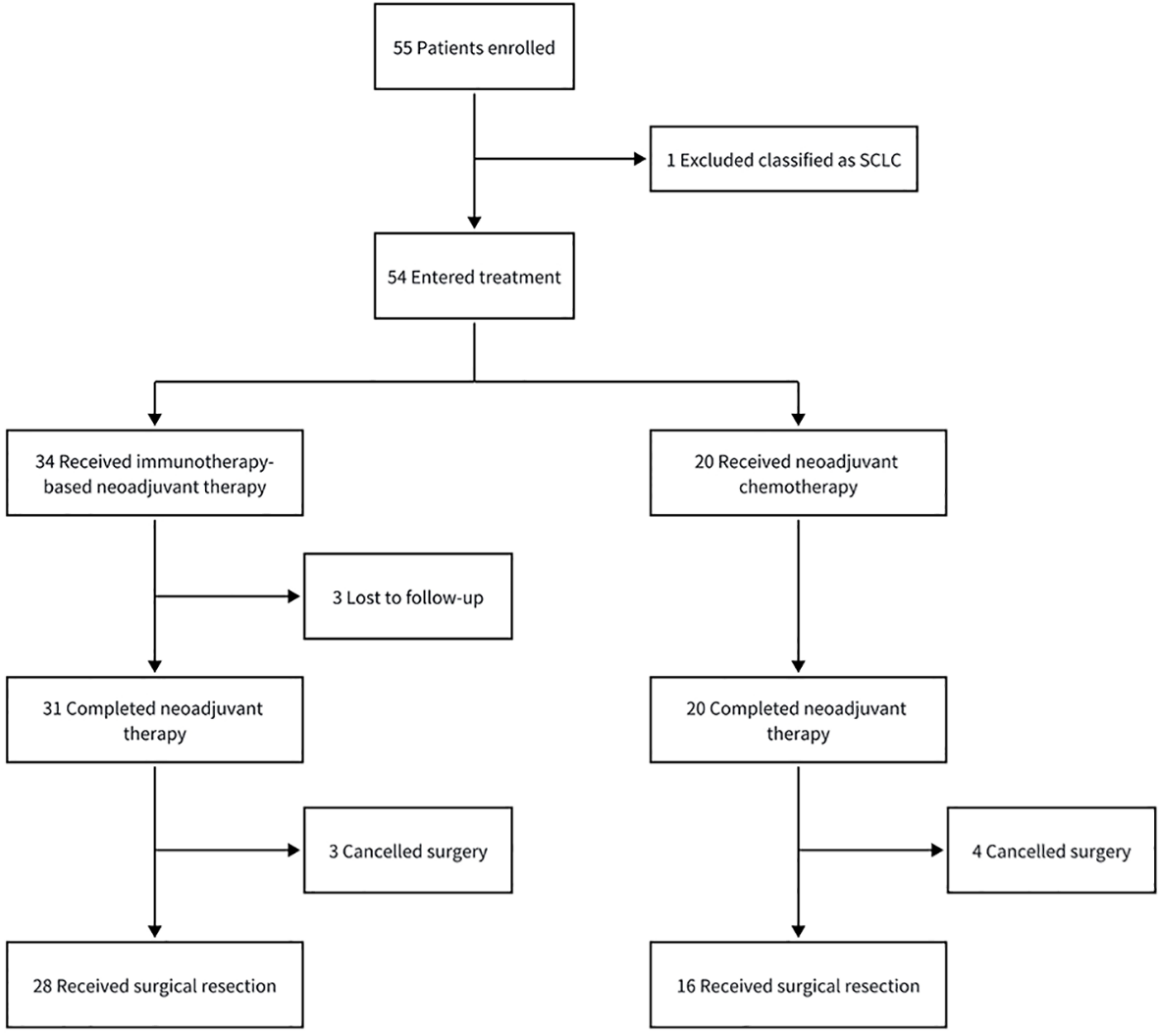
Study profile. SCLC, small cell lung cancer.

From December 2019 through July 2022, a total of 51 patients completed neoadjuvant therapy (**Figure 1**). Of these, 31 patients received immunotherapy-based neoadjuvant therapy, and 20 patients received neoadjuvant chemotherapy alone (**Table 1**).

**Table 1:** Characteristics of patients at baseline.

	Immunotherapy* (N = 31)	Chemotherapy (N = 20)
Age at enrollment: years		
Median (IQR)	61 (53-65)	63.5 (56.5-68.75)
Sex: no. (%)		
Male	26 (83.9)	17 (85)
Female	5 (16.1)	3 (15)
ECOG performance status: no. (%)		
0	2 (6.5)	0
1	29 (93.5)	20 (100)
Smoking status: no. (%)		
Never	12 (38.7)	6 (30)
Former or current	19 (61.3)	14 (70)
Histology: no. (%)		
Adenocarcinoma	8 (25.8)	8 (40)
Squamous cell carcinoma	20 (64.5)	12 (60)
NSCLC NOS	3 (9.7)	0
Disease stage: no. (%)**		
IB or IIB	3 (9.7)	1 (5)
IIIA	12 (38.7)	10 (50)
IIIB	16 (51.6)	8 (40)
IIIC	0	1 (5)
Baseline T: no. (%)		
T1b	1 (3.2)	1 (5)
T1c	2 (6.5)	1 (5)
T2a	6 (19.4)	1 (5)
T2b	4 (12.9)	5 (25)
T3	6 (19.4)	6 (30)
T4	12 (38.7)	6 (30)
Baseline N: no. (%)		
N0	2 (6.5)	0
N1	4 (12.9)	4 (20)
N2	24 (77.4)	15 (75)
N3	1 (3.2)	1 (5)
PD-L1 expression level: no. (%)		
<1%	10 (32.3)	4 (20)
≥1%	13 (41.9)	4 (20)
1–49%	6 (19.4)	2 (10)
≥50%	7 (22.6)	2 (10)
Unknown	8 (25.8)	12 (60)

Data are n (%) or median (IQR). ECOG, Eastern Cooperative Oncology Group; NSCLC, non–small cell lung cancer; NOS, not otherwise specified; IQR, interquartile range.

* Patients in the immunotherapy group received immunotherapy-based neoadjuvant therapy.

** The disease stage was evaluated according to the staging criteria of the American Joint Committee on Cancer, 8th edition.

Among the patients receiving immunotherapy-based neoadjuvant therapy, 27 received immunotherapy plus chemotherapy (25 of these patients received immunotherapy plus platinum-based chemotherapy), one received pembrolizumab alone, one received sintilimab alone, and two received nivolumab plus ipilimumab. Except for two patients who received only one cycle of neoadjuvant immunotherapy owing to adverse events, the majority of patients received two to three cycles of neoadjuvant immunotherapy (**Table S1**).

### 3.2 Surgery

In total, 90.3% of patients receiving immunotherapy-based neoadjuvant therapy and 80.0% of patients receiving neoadjuvant chemotherapy ultimately underwent surgical resection. Surgery was canceled for three patients in the immunotherapy group and four patients in the chemotherapy group. Because the radiologic response to three cycles of neoadjuvant chemotherapy is complete, one patient canceled the surgery. Another patient in the immunotherapy group canceled the surgery because of disease progression. Another three chemotherapy patients and two immunotherapy patients were found to be unresectable by multidisciplinary teams. In total, 96.4% of patients in the immunotherapy group and 93.8% of patients in the chemotherapy group underwent R0 resection (no residual tumor) (**Table 2**).

**Table 2:** Surgical outcomes.

	Immunotherapy (N = 31)	Chemotherapy (N = 20)
Patients with surgical resection: no. (%)	28 (90.3)	16 (80)
Patients with canceled surgery: no. (%)	3 (9.7)	4 (20)
Complete response	0	1 (5)
Disease progression	1 (3.2)	0
Unresectable	2 (6.5)	3 (15)
Time from last neoadjuvant dose to surgical resection: weeks		
Median (IQR)	4.8 (3.9-5.3)	4.6 (3.9-5.0)
Surgical approach: no. (%)		
Thoracotomy	21 (75)	10 (62.5)
Minimally invasive*	7 (25)	6 (37.5)
Type of surgery: no. (%)		
Lobectomy	16 (57.1)	13 (81.3)
Sleeve lobectomy	8 (28.6)	2 (12.5)
Bilobectomy	3 (10.7)	1 (6.3)
Pneumonectomy	1 (3.6)	0
Estimated blood loss: mL		
Median (IQR)	100 (100-200)	150 (100-200)
Length of hospital stay after surgery: days		
Median (IQR)	6 (5-10)	6 (4-8)
Patients transferred to the ICU after surgical resection: no. (%)	14 (50)	6 (37.5)
Length of ICU stay after surgery: days		
Median (IQR)	1 (1-1.3)	2 (1-3)
Completeness of resection: no. (%)**		
R0 (no residual tumor)	27 (96.4)	15 (93.8)
R1 (microscopic residual tumor)	1 (3.6)	1 (6.3)
R2 (macroscopic residual tumor)	0	0
Sampled lymph nodes		
Median (IQR)	16 (9-25)	19 (13-26)
Postoperative complications		
30-day mortality	0	1 (5)
30- to 90-day mortality	0	0
Pneumonia	1 (3.6)	1 (5)
Wound infection	2 (6.5)	0
Chylothorax	1 (3.6)	0
Pneumothorax	1 (3.6)	1 (5)
Hypoxemia	0	1 (5)
Pain	1 (3.6)	0

Data are n (%) or median (IQR). ICU, intensive care unit.

* Thoracoscopic/robotic.

** R0 and R1 operation rates were calculated in the surgical population.

Postoperative complications are summarized in **Table 2**. One patient died of hypoxemia two weeks after surgery; however, this was deemed to have been unrelated to the study treatments.

### 3.3 Efficacy

**Figure 2:**
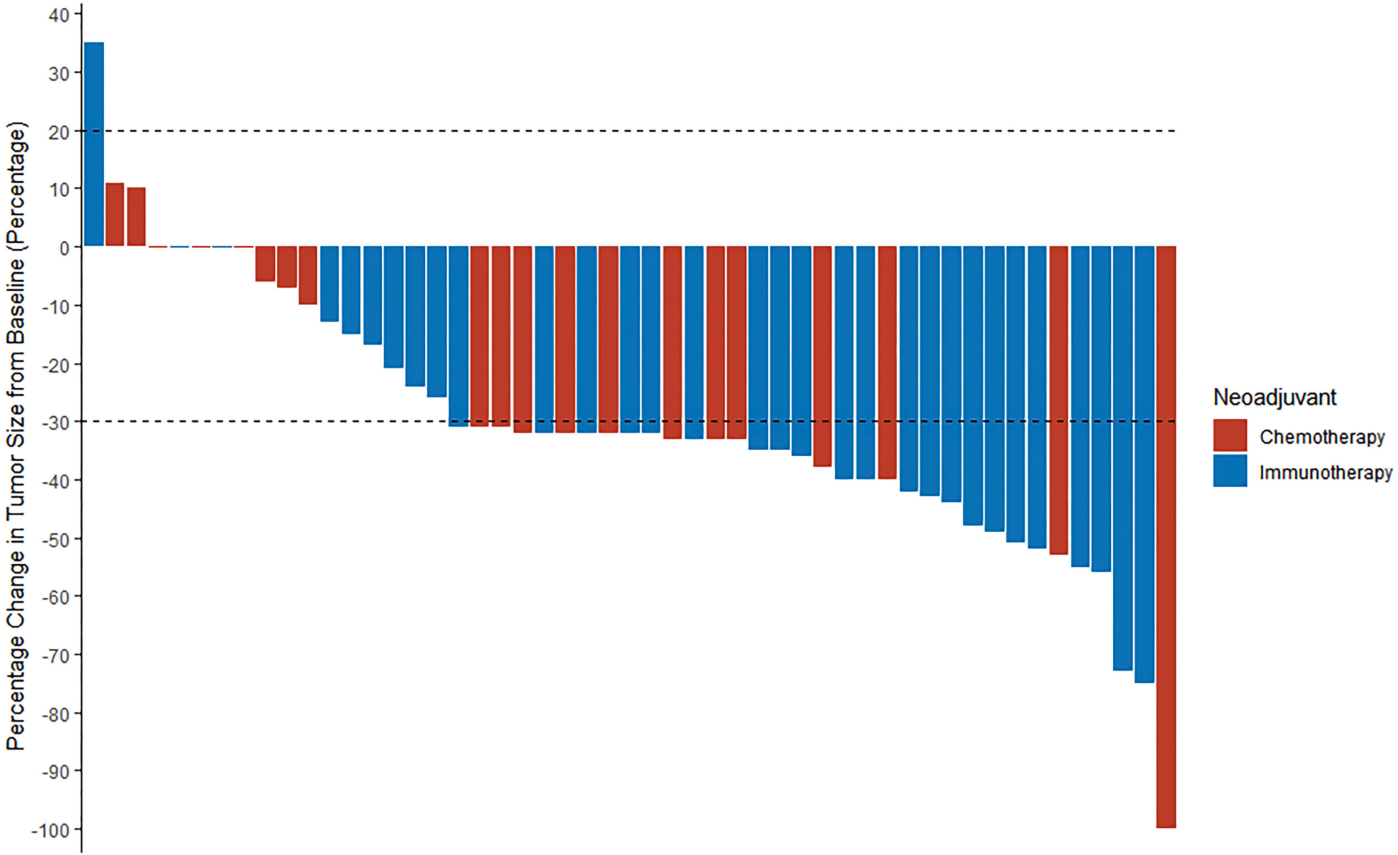
A waterfall plot of radiologic response in all patients (n = 51). The two dashed lines are the standard lines for PR (-30%) and PD (20%), respectively. PR, partial response; PD, progressive disease.

After immunotherapy-based neoadjuvant therapy, the TRR was 71.0% (22 of 31; 95% CI, 0.534 to 0.839), all of whom achieved a PR. After neoadjuvant chemotherapy, the TRR according to RECIST 1.1 was 60.0% (12 of 20; 95% CI, 0.387 to 0.781; *P* = 0.417), including one (5%) patient with a CR and 11 (55%) patients with a PR (**Figures 2**; **Figure S1**). Radiographic downstaging before resection occurred in 15 (48.4%) patients in the immunotherapy group and seven (35%) patients in the chemotherapy group.

Among all the patients regardless of resection, the percentage with a pathologic complete response was 19.4% (6 of 31; 95% CI, 0.092 to 0.363) with immunotherapy-based neoadjuvant therapy and 5% (1 of 20; 95% CI, 0.009 to 0.236; *P* = 0.223) with neoadjuvant chemotherapy alone. Compared to chemotherapy, immunotherapy had a much higher percentage of patients who had a major pathologic response (41.9% vs. 15.0%; 95% CI, 0.008 to 0.468; *P* = 0.043) (**Figure S2**). In the subgroup analysis of patients in the immunotherapy group, 50% of patients with PD-L1 < 1% achieved a major pathological response, and 23.1% of patients with PD-L1 ≥ 1% achieved a major pathological response (*P* = 0.221). Among the patients who underwent surgical resection, pathologic downstaging from the pretreatment clinical stage occurred in 58.1% (18 of 28) of patients with immunotherapy-based neoadjuvant therapy and 50% (10 of 16) of patients with neoadjuvant chemotherapy alone.

Our immunotherapy-based neoadjuvant therapy protocol includes several different treatment modalities. The radiologic response to neoadjuvant therapy was 84.0% in patients receiving immunotherapy plus chemotherapy and 16.7% (*P* = 0.004) in those receiving other types of immunotherapy. The percentage of patients achieving a major pathological response was 44.0% with immunotherapy plus chemotherapy and 33.3% with other types of immunotherapy (**Table S2**).

The radiologic response to neoadjuvant therapy was 83.3% in patients who received two to three cycles of neoadjuvant immunotherapy plus chemotherapy. The percentage of patients achieving a major pathologic response was 25.0% with two cycles of neoadjuvant immunotherapy plus chemotherapy and 66.7% (*P* = 0.100) with three cycles. The percentage of patients achieving a complete pathologic response was 25% and 33.3%, respectively (**Table S2**).

**Figure 3:**
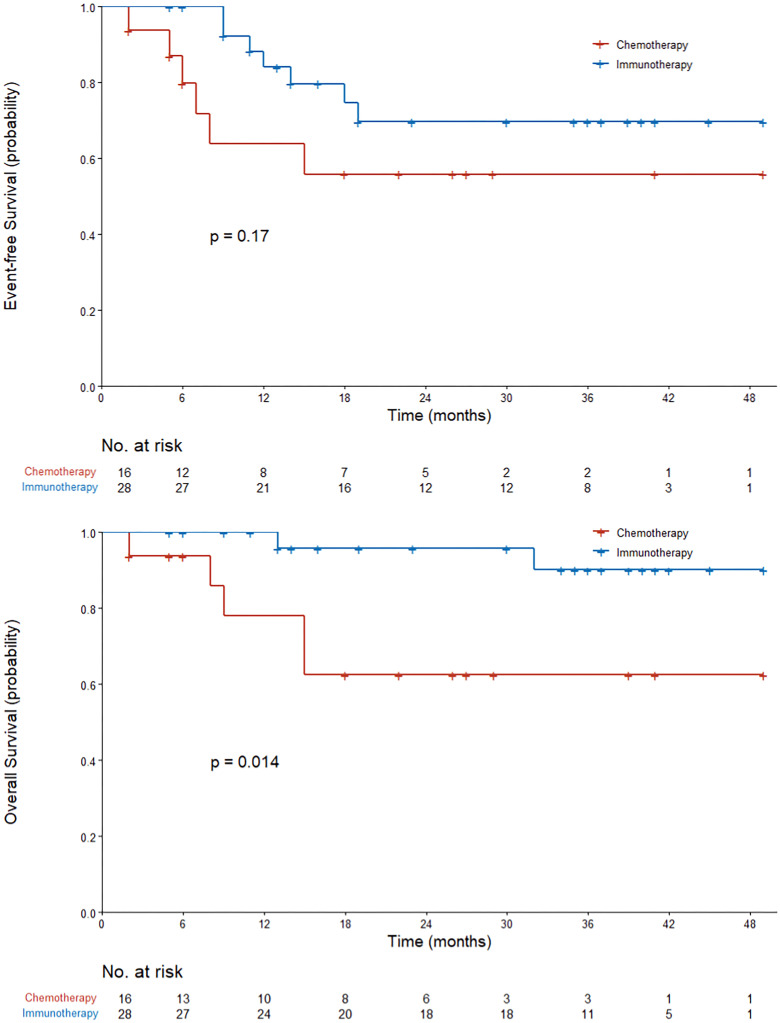
Kaplan–Meier curves of event-free survival and overall survival in all the patients who received neoadjuvant therapy. The P value was calculated using the log-rank test.

The survival and recurrence of the 44 patients who underwent surgical resection were tracked. At a median follow-up of 28 months from the first dose of neoadjuvant therapy, 21 of 28 patients (75%) in the immunotherapy group were alive and recurrence-free and 10 of 16 patients (62.5%) in the chemotherapy group. The median duration of EFS and OS was not reached in either group. Neoadjuvant immunotherapy patients had a significantly better OS compared to chemotherapy patients (log-rank *P* = 0.014). In the immunotherapy group, EFS was 84.1% at 12 months and 74.7% at 18 months. In the chemotherapy group, EFS was 63.8% at 12 months and 55.9% at 18 months. In the immunotherapy group, OS was 100% at 12 months and 95.8% at 18 months. In the chemotherapy group, OS was 78.1% at 12 months and 62.5% at 18 months (**Figure 3**).

### 3.4 Safety

Adverse events occurred in 64.5% of patients in the immunotherapy group and 55% of patients in the chemotherapy group during neoadjuvant therapy. The most common adverse events of any grade were a decrease in neutrophil count (32.3% in the immunotherapy group and 45% in the chemotherapy group) and a decrease in white blood cell count (32.3% and 40%, respectively). The most common adverse event in grades 3 to 4 was a decrease in neutrophil count (22.6% in the immunotherapy group and 30% in the chemotherapy group). None of the adverse events occurred during neoadjuvant therapy resulted in death or a delay in surgery (**Table 3**).

**Table 3:** Adverse events during neoadjuvant therapy.

Adverse events: no. (%)	Chemotherapy (N = 20)			Immunotherapy (N = 31)		
	Any Grade	Grade 3	Grade 4	Any Grade	Grade 3	Grade 4
Any adverse event	11 (55)	6 (30)	3 (15)	20 (64.5)	7 (22.6)	1 (3.2)
Neutrophil count decreased	9 (45)	3 (15)	3 (15)	10 (32.3)	6 (19.4)	1 (3.2)
White blood cells decreased	8 (40)	3 (15)	0	10 (32.3)	1 (3.2)	0
Platelet count decreased	2 (10)	1 (5)	0	4 (12.9)	0	0
Alanine aminotransferase increased	1 (5)	0	0	1 (3.2)	0	0
Rash	1 (5)	0	0	3 (9.7)	0	0
Nausea	0	0	0	3 (9.7)	0	0
Vomiting	0	0	0	2 (6.5)	1 (3.2)	0
Anemia	0	0	0	2 (6.5)	0	0
Febrile neutropenia	0	0	0	1 (3.2)	1 (3.2)	0
Mucositis oral	0	0	0	1 (3.2)	0	0
Pneumonitis	0	0	0	1 (3.2)	0	0
Myocardial infarction	0	0	0	1 (3.2)	0	0

Data are n (%). Adverse events were graded according to Common Terminology Criteria for Adverse Events, version 4.0. No grade 5 adverse events were reported.

## 4 Discussion

In this observational clinical study, the proportion of patients achieving a major pathologic response with two to three cycles of immunotherapy-based neoadjuvant therapy significantly increased from 15.0% to 41.9% (*P* = 0.043) compared to chemotherapy alone, but such a significant increase was not found in the proportion of patients achieving a pathologic complete response (19.4% vs. 5%; 95% CI, -0.069 to 0.318; *P* = 0.223) with immunotherapy-based neoadjuvant therapy. At the time of data cutoff (October 31, 2022), we found that patients in the immunotherapy group had better survival outcomes than those in the chemotherapy group (log-rank *P* = 0.014). In addition, in other study endpoints—including TRR, event-free survival, radiographic downstaging, and pathologic downstaging—we found a benefit of immunotherapy-based neoadjuvant therapy versus neoadjuvant chemotherapy alone, but the difference was not significant. Adverse events during neoadjuvant therapy in the two treatment groups were manageable and did not differ significantly.

By analyzing the surgical approach, we observed a high rate of thoracotomy in two treatment groups, and the rate in immunotherapy patients was higher than that in chemotherapy patients (75% vs. 62.5%, *P* = 0.496). According to recent studies, a low tensile strength of the pulmonary vessels and bronchi was found in patients receiving neoadjuvant chemotherapy (18), and more severe destruction of elastic fibers of the blood vessels, pulmonary interstitial exudation, vascular wall degeneration, fibrinoid necrosis, and fibrosis was found in patients receiving neoadjuvant immunotherapy (19). Such histological changes could explain the high rate of thoracotomy in two treatment groups. Also, we evaluated the difficulty of the surgery by type of surgery; estimated blood loss; length of hospital stay after surgery; whether transfer to the ICU was required, and if so, the length of the ICU stay. We did not find significant differences between the immunotherapy group and the chemotherapy group in any of these indexes. After surgical resection, one patient who received neoadjuvant chemotherapy died of postoperative complication (hypoxemia) within 30 d. In both the immunotherapy and chemotherapy groups, we observed a lower proportion of postoperative complications compared with other relevant observational studies (19–21). This might be because we performed the postoperative complication statistics only from the medical records during the hospital stay after surgery, which would lead to errors. We did not find a significant difference between the two treatment groups only through the available statistics of postoperative complications. Overall, treatment with immunotherapy-based neoadjuvant therapy did not increase the complexity or difficulty of surgical resection and the possibility of postoperative complications.

Many previous studies set OS and EFS as the primary endpoints to determine the clinical effectiveness of drug or treatment modalities, but this approach can be time consuming and thus may impede the marketing and promotion of clinical drugs. Several studies (11, 13, 22) have shown that a pathologic response is strongly correlated with clinical survival. Clinical studies NCT02716038 (12) and NEOSTAR (23) set MPR as the primary endpoint. Furthermore, the clinical study CheckMate 816 (11) innovatively set pCR as the primary endpoint and found a strong association between pCR and clinical benefit. Considering the small sample size of our study, which resulted in a low incidence of pCR, we finally set MPR as the primary endpoint for *post hoc* subgroup analysis.

In most subgroups, we discovered a relative benefit of immunotherapy-based neoadjuvant therapy compared to neoadjuvant chemotherapy by assessing the pathologic response in subgroups. Patients who smoked showed a greater benefit than those who had never smoked. Previous reports have also shown that smokers are more likely to benefit from T-cell checkpoint blockade (24, 25). Additionally, patients with stage III disease experienced a greater benefit than those with stage I or stage II disease, which was also seen in the CheckMate 816 clinical study (11). A pooled analysis of lung adjuvant cisplatin found that the therapeutic benefit varies by stage and is greatest for patients with stages II and III (26). To demonstrate that patients with stage III disease have a better prognosis from participating in the immunotherapy group, a longer postoperative follow-up period may be needed. In addition, only a small number of patients with stage I or stage II disease were included in our study (one with neoadjuvant chemotherapy and three with neoadjuvant immunotherapy), which might have undermined the statistical value of our findings. According to our findings, major pathologic response and tumor PD-L1 expression were not significantly correlated (50% in patients with PD-L1 < 1%; 23.1% in patients with PD-L1 ≥ 1%; *P* = 0.221), which indicates that PD-L1 expression cannot predict treatment benefit.

Treatments in the immunotherapy group in our study included multiple modalities, and we tried to determine whether different treatment modalities could result in different prognoses. We found a greater benefit, in terms of MPR and pCR, from immunotherapy plus chemotherapy versus other types of immunotherapy, but this was not significant. In terms of TRR, the benefit in patients receiving immunotherapy plus chemotherapy was significant. Among all the patients who received immunotherapy, an increase was found—from 26.7% to 64.3% (*P* = 0.066)—in the proportion of those achieving a major pathologic response with three cycles of neoadjuvant therapy versus only two cycles, although this was not significant. Encouragingly, there was also no significant increase in adverse events during neoadjuvant therapy. We still need a larger sample size to clarify whether increasing the number of neoadjuvant cycles significantly improves efficacy, but at least we did not observe any negative treatment outcomes. In addition, we mainly analyzed pembrolizumab and nivolumab, which are commonly used in patients with NSCLC in China. We did not find a significant difference between pembrolizumab and nivolumab in terms of MPR, pCR, or TRR or in adverse events. Although, because of the CheckMate 816 clinical study (11), the US Food and Drug Administration has approved nivolumab plus platinum-based chemotherapy for neoadjuvant therapy in NSCLC patients (27), there are very few clinical studies of pembrolizumab for the treatment of NSCLC. With reference to the results of our study, we think that pembrolizumab may have promising efficacy and safety similar to that of nivolumab for neoadjuvant therapy of NSCLC.

Overall, this observational study confirmed that in resectable NSCLC, immunotherapy-based neoadjuvant therapy offered a significantly greater benefit in terms of major pathologic response and did not enhance the risk of adverse events or hinder surgical resection. Our study has limitations, including a limited sample size and a short postoperative follow-up period. We therefore hope to extend it in the future with a larger sample of patients and longer follow-up to further elucidate the clinical benefits of our approach over 5 and 10 years.

## Data availability statement

The raw data supporting the conclusions of this article will be made available by the authors, without undue reservation.

## Ethics statement

The studies involving humans were approved by the Institutional Review Board of Shang Chest Hospital (No. KS1971). The studies were conducted in accordance with the local legislation and institutional requirements. The participants provided their written informed consent to participate in this study.

## Author contributions

JS: Data curation, Formal Analysis, Investigation, Resources, Validation, Visualization, Writing – original draft, Writing – review & editing. LG: Conceptualization, Data curation, Methodology, Validation, Writing – review & editing. YQ: Data curation, Investigation, Validation, Writing – review & editing. YY: Methodology, Supervision, Writing – review & editing. SL: Conceptualization, Methodology, Project administration, Supervision, Validation, Writing – review & editing. ZC: Funding acquisition, Methodology, Project administration, Supervision, Validation, Writing – review & editing.
