# Supplementary material for: Real-world outcomes of immunotherapy-based neoadjuvant therapy in resectable non-small cell lung cancer
Source: Front Immunol. 2023 Sep 25;14:1268251. doi: 10.3389/fimmu.2023.1268251 (PMC10560710; doi:10.3389/fimmu.2023.1268251)
Supplement: Supplementary file 1 [file DataSheet_1.docx]

Table S1.

Modalities of Neoadjuvant Immunotherapy

|  | Immunotherapy  (N = 31) |
| --- | --- |
| Neoadjuvant therapy: no. (%)* |  |
| Immunotherapy + chemotherapy | 27(87.1) |
| Platinum-based chemotherapy | 25(80.6) |
| Other chemotherapy^ | 2(6.5) |
| Mono-immunosuppressive therapy$ | 2(6.5) |
| Nivolumab + ipilimumab | 2(6.5) |
| Type of PD-1 inhibitor: no. (%)** |  |
| Pembrolizumab | 13(52) |
| Nivolumab | 5(20) |
| Sintilimab | 5(20) |
| Tislelizumab | 2(8) |
| Cycle of neoadjuvant therapy: no. (%)*** |  |
| 1# | 2(6.5) |
| 2 | 15(48.4) |
| 3 | 14(45.2) |

* The number and percentage of each type of neoadjuvant therapy were calculated in the population of patients receiving neoadjuvant immunotherapy.

** The number and percentage of the types of PD-1 inhibitors were calculated in the population of patients receiving neoadjuvant immunotherapy plus platinum-based chemotherapy.

*** The number and percentage of each type of neoadjuvant therapy cycle were calculated in the population of patients receiving neoadjuvant immunotherapy.

$ Patients receiving mono-immunosuppressive therapy included 1 patient who received pembrolizumab and 1 patient who received sintilimab.

^ Other chemotherapy in patients receiving immunotherapy with chemotherapy included docetaxel and nab-paclitaxel.

# 1 patient received one cycle of tislelizumab + platinum-based chemotherapy and then stopped neoadjuvant immunotherapy due to adverse events and finally underwent surgical resection directly when protocol-defined criteria for treatment resumption were met; the other patient received one dose of sintilimab and then stopped neoadjuvant immunotherapy due to adverse events and then underwent radiation therapy.

Table S2.

Efficacy of Different Neoadjuvant Immunotherapy Modalities

|  | TRR | MPR | pCR |
| --- | --- | --- | --- |
| Neoadjuvant immunotherapy |  |  |  |
| Immunotherapy + platinum-based chemotherapy | 84.0% | 44.0% | 28.0% |
| Other immunotherapy | 16.7% | 33.3% | 16.7% |
| *P* value | 0.004 | 1.000 | 1.000 |
| Cycles of neoadjuvant therapy* |  |  |  |
| 2 | 83.3% | 25.0% | 25.0% |
| 3 | 83.3% | 66.7% | 33.3% |
| *P* value | 1.000 | 0.100 | 1.000 |
| Type of PD-1 inhibitor* |  |  |  |
| Pembrolizumab | 92.3% | 46.2% | 38.5% |
| Nivolumab | 60.0% | 60.0% | 0 |
| *P* value | 0.172 | 1.000 | 0.249 |

Abbreviations: TRR, radiologic response to neoadjuvant therapy. MPR, major pathologic response. pCR, complete pathologic response.

* Cycle of neoadjuvant therapy and type of PD-1 inhibitor were both calculated in patients with immunotherapy plus platinum-based chemotherapy.

Figure S1.

Radiologic response in patients with neoadjuvant chemotherapy and immunotherapy, respectively


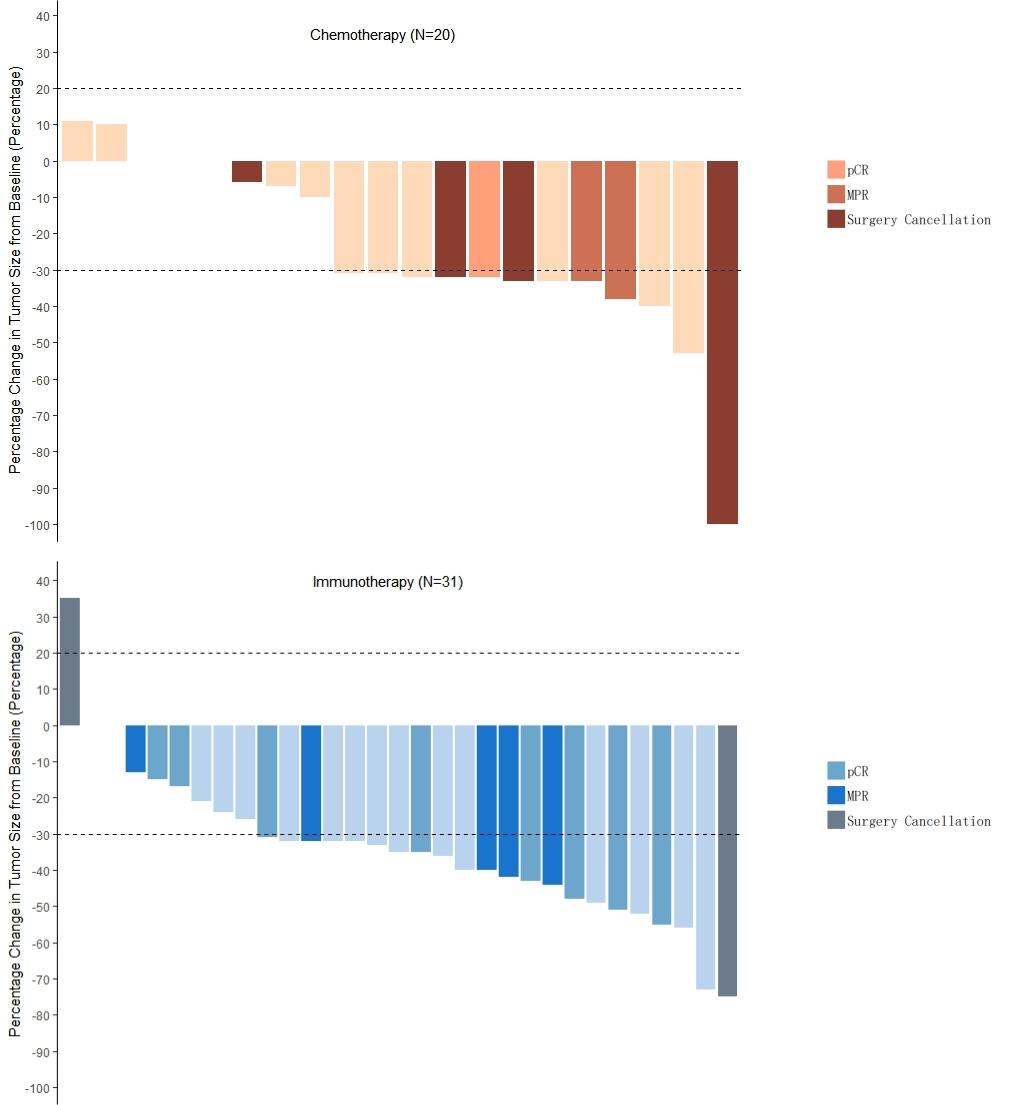


Fig. S1 was waterfall plot of radiologic response in patients with neoadjuvant chemotherapy and immunotherapy, respectively. The two dashed lines are the standard lines for PR (-30%) and PD (20%), respectively.

Figure S2

Major pathologic response in prespecified patient subgroups


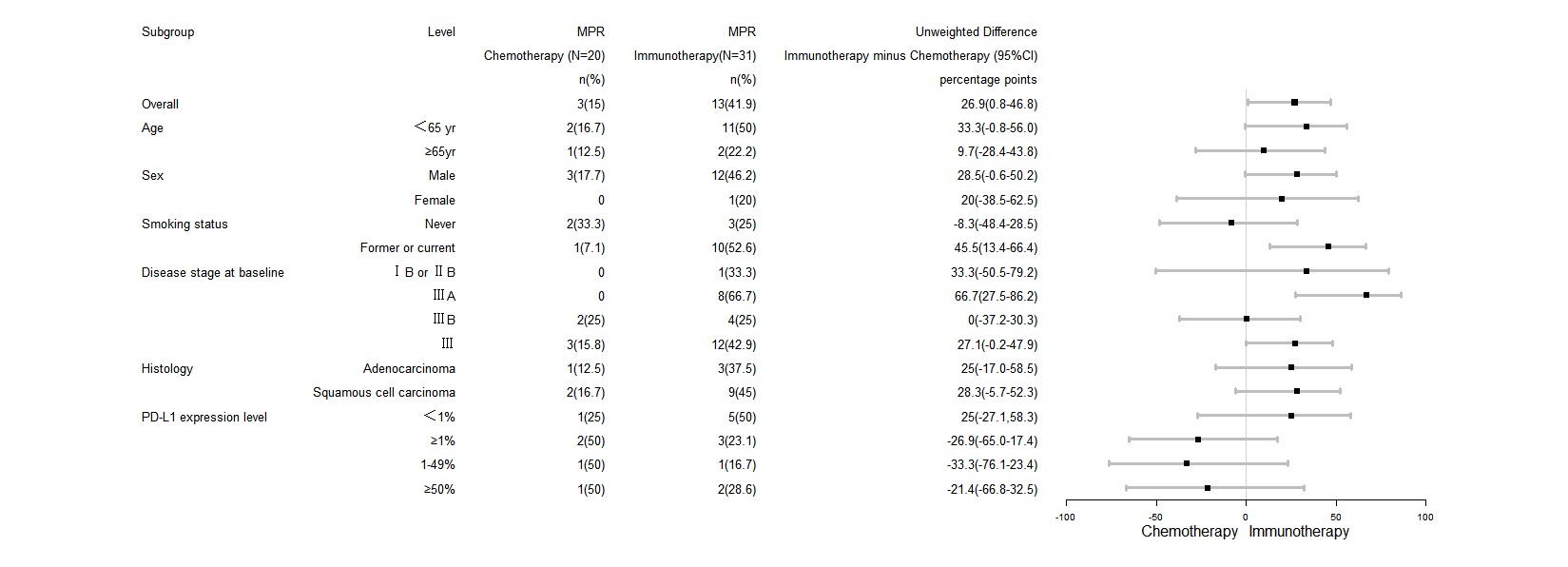


Figure S2 shows Major pathologic response (MPR), or ≤ 10% residual viable tumor cells in the primary tumor, in prespecified patient subgroups. Patients who did not undergo surgical resection were counted as not having a major pathologic response.
